# Supplementary material for: Ant collective cognition allows for efficient navigation through disordered environments
Source: eLife. 2020 May 12;9:e55195. doi: 10.7554/eLife.55195 (PMC7332297; doi:10.7554/eLife.55195)
Supplement: Transparent reporting form [file elife-55195-transrepform.pdf]

Westbrook Centre, Milton Road
Cambridge CB4 1YG
UK
P 01223 855340
W elifesciences.org
T @elife

eLife's transparent reporting form

We encourage authors to provide detailed information *within their submission* to facilitate the interpretation and replication of experiments. Authors can upload supporting documentation to indicate the use of appropriate reporting guidelines for health-related research (see EQUATOR Network), life science research (see the BioSharing Information Resource), or the ARRIVE guidelines for reporting work involving animal research. Where applicable, authors should refer to any relevant reporting standards documents in this form.

If you have any questions, please consult our Journal Policies and/or contact us: editorial@elifesciences.org.

Sample-size estimation

- You should state whether an appropriate sample size was computed when the study was being designed
- You should state the statistical method of sample size computation and any required assumptions
- If no explicit power analysis was used, you should describe how you decided what sample (replicate) size (number) to use

Please outline where this information can be found within the submission (e.g., sections or figure legends), or explain why this information doesn't apply to your submission:

Experiments were conducted during the summer of 2015 and 2018. The only time in which this species exhibits cooperative transport is a period of about 3 months during the summer. We initially decided on the experiments required and used the available time to conduct as many replicates as possible of each experiment before the summer is over. Data from all relevant experiments was then included in the paper. Statistical significance is specified where appropriate.

Replicates

- You should report how often each experiment was performed
- You should include a definition of biological versus technical replication
- The data obtained should be provided and sufficient information should be provided to indicate the number of independent biological and/or technical replicates
- If you encountered any outliers, you should describe how these were handled
- Criteria for exclusion/inclusion of data should be clearly stated
- High-throughput sequence data should be uploaded before submission, with a private link for reviewers provided (these are available from both GEO and ArrayExpress)

Please outline where this information can be found within the submission (e.g., sections or figure legends), or explain why this information doesn't apply to your submission:

We excluded all experiments where the ants were able to solve the maze by climbing over the cubes before the allotted maximum time of 8 minutes passed, except for calculation of maze crossing success probability, where these were treated as failed attempts.

A penalty was defined for the calculation of arc-length for failed attempts, as described in figure 2c caption.

All replicates were biological in nature. A biological replicate consisted of running an experiment over a maze of equal density but not the same cube configuration. The cube configuration was randomized for each replicate.

Cube and trajectory data are provided within the full submission.

Information regarding sample sizes of the different experiments and simulations is specified in a dedicated supplementary section A.23: Sample Sizes.

Statistical reporting

- Statistical analysis methods should be described and justified
- Raw data should be presented in figures whenever informative to do so (typically when N per group is less than 10)
- For each experiment, you should identify the statistical tests used, exact values of N, definitions of center, methods of multiple test correction, and dispersion and precision measures (e.g., mean, median, SD, SEM, confidence intervals; and, for the major substantive results, a measure of effect size (e.g., Pearson's r, Cohen's d)
- Report exact p-values wherever possible alongside the summary statistics and 95% confidence intervals. These should be reported for all key questions and not only when the p-value is less than 0.05.

Please outline where this information can be found within the submission (e.g., sections or figure legends), or explain why this information doesn't apply to your submission:

Information regarding error margins is provided in figure captions: 2b-c, 3c-d, 4a-b. Generally we used standard error of the mean wherever we wanted to compare the results of the different simulations and the ants, and plain standard deviation when we wanted to show the spread of the data. In all of these cases we used means of the data as our measure. Whether standard error of the mean or standard deviation was used in every individual case is clearly stated in the corresponding figure caption.

Statistical test and their results are reported in the following supplementary sections:
A.6: Rolling behavior upon encountering single cubes.
A.10: Comparing experimental and simulated backward motion characteristics of the load when it is trapped.
A.14: Comparing motion characteristics of the load in single entrance vs. composite traps.

(For large datasets, or papers with a very large number of statistical tests, you may upload a single table file with tests, Ns, etc., with reference to sections in the manuscript.)

Group allocation

- Indicate how samples were allocated into experimental groups (in the case of clinical studies, please specify allocation to treatment method); if randomization was used, please also state if restricted randomization was applied
- Indicate if masking was used during group allocation, data collection and/or data analysis

Please outline where this information can be found within the submission (e.g., sections or figure legends), or explain why this information doesn't apply to your submission:

Experimental grouping of experiments was done by the number of cubes used to create the mazes.

Additional data files ("source data")

- We encourage you to upload relevant additional data files, such as numerical data that are represented as a graph in a figure, or as a summary table
- Where provided, these should be in the most useful format, and they can be uploaded as "Source data" files linked to a main figure or table
- Include model definition files including the full list of parameters used
- Include code used for data analysis (e.g., R, MatLab)
- Avoid stating that data files are "available upon request"

Please indicate the figures or tables for which source data files have been provided:

We provide the data for the experimental trajectories as well as the cube mazes used throughout the article.
